# The genotypic spectrum of complex febrile seizures: insights from high-risk population genetic screening in a pediatric cohort

**DOI:** 10.3389/fnins.2026.1828448

**Published:** 2026-06-05

**Authors:** Xiaolong Deng, Yan Xu, Xue Chen, Juan Pan, Sheng Huang, Dan Sun

**Affiliations:** 1 Department of Neurology, Wuhan Children’s Hospital, Tongji Medical College, Huazhong University of Science and Technology, Wuhan, Hubei, China; 2 Department of Child Health Care, Wuhan Jiang’an District Maternal and Child Health Hospital, Wuhan, Hubei, China

**Keywords:** complex febrile seizures, Dravet syndrome, genotype–phenotype correlation, ion channelopathy, pediatric epilepsy, precision medicine, SCN1A, targeted gene panel sequencing

## Abstract

**Background:**

Complex febrile seizures (CFS) confer an elevated risk of epilepsy progression; however, the underlying genetic architecture remains insufficiently characterized in Chinese pediatric populations. This study aimed to delineate the mutational landscape and genotype–phenotype associations in a clinically stratified high-risk febrile seizure cohort.

**Methods:**

This retrospective, single-center study enrolled 233 children (aged 6 months–6 years) who were consecutively screened at the Wuhan Children’s Hospital (July 2019–January 2025) and fulfilled ≥ 1 predefined high-risk criterion. Targeted epilepsy gene panel sequencing was performed, and variant pathogenicity was adjudicated according to ACMG/AMP guidelines. Between-group comparisons were made using the Mann–Whitney U test, Pearson’s χ^2^ test with continuity correction, or Fisher’s exact test; effect sizes are reported as odds ratios (OR) with 95% confidence intervals (CI).

**Results:**

Sixty-seven patients (28.8%) harbored pathogenic/likely pathogenic (P/LP) variants in 18 genes. Voltage-gated sodium channel genes (SCN1A, SCN1B, SCN2A, SCN8A) accounted for 44.8% of positive cases, with SCN1A being most prevalent (25.4%). Patients fulfilling ≥ 2 high-risk criteria demonstrated a higher diagnostic yield than those with a single criterion (35.4% vs. 20.8%; OR = 2.10; 95% CI: 1.16–3.79; *p* = 0.020). P/LP-positive patients exhibited significantly elevated rates of status epilepticus (OR = 4.96), developmental delay (OR = 3.70), and abnormal interictal EEG (OR = 3.03). Among SCN1A-positive patients, 70.6% progressed to Dravet syndrome. Genetic findings modified antiseizure medication management in 62.7% of positive cases.

**Conclusion:**

Targeted genetic screening in high-risk CFS populations yields a clinically significant diagnostic rate dominated by ion channel genes, facilitating early epilepsy risk identification and precise therapeutic intervention.

## 1 Introduction

Febrile seizures (FS) constitute the most prevalent convulsive disorder of early childhood, affecting an estimated 2%–5% of children aged 6 months to 5 years in Western populations and a higher proportion (ranging from 4.3% to 6.9 %) in East Asian cohorts (Chung, 2014; Eilbert and Chan, 2022; Leung et al., 2018). The International League Against Epilepsy (ILAE) classifies FS into simple and complex subtypes; the latter is characterized by a prolonged duration exceeding 15 min, focal or lateralizing semiology, or recurrence within a single febrile episode (Steering Committee on Quality Improvement Management Subcommittee on Febrile Seizures American Academy of Pediatrics, 2008). In contrast to simple FS, which are overwhelmingly self-limited and confer a negligible long-term epilepsy risk of 1%–2%, complex FS (CFS) occupies a distinct pathophysiological niche: epidemiological investigations consistently demonstrate that 4%–15% of children with CFS subsequently develop epilepsy, representing a 3- to 8-fold relative risk elevation compared to the general pediatric population (Baso et al., 2025; Lee et al., 2016; Tsai et al., 2018; Verity and Golding, 1991).

The pathogenesis of febrile seizure-to-epilepsy transition involves a complex interplay of extrinsic environmental triggers, principally infection-induced pyrexia and associated neuroinflammatory cascades, and intrinsic neurobiological vulnerabilities, including age-dependent neuronal hyperexcitability attributable to incomplete axonal myelination and immature γ-aminobutyric acid (GABA)-ergic inhibitory circuitry (Dimitrijevic et al., 2018; Kang and Macdonald, 2016; Kwon et al., 2018; Wu et al., 2025). A substantial heritable component has been established through twin concordance studies, which have demonstrated rates of 56%–70% in monozygotic pairs compared to 14%–18% in dizygotic pairs, and through genome-wide association studies implicating loci involved in thermoregulatory responses, synaptic transmission, and neuronal excitability (Audenaert et al., 2006; Skotte et al., 2022).

Among the monogenic determinants of fever-sensitive epilepsy, voltage-gated ion channel genes have emerged as the most clinically consequential. SCN1A, which encodes the Nav1.1 α-subunit of the voltage-gated sodium channel, underlies a phenotypic continuum spanning genetic epilepsy with febrile seizures plus (GEFS+) to Dravet syndrome, a severe developmental and epileptic encephalopathy characterized by treatment-resistant seizures, cognitive regression, and elevated mortality (Claes et al., 2001; Scheffer and Nabbout, 2019; Wirrell et al., 2017). The identification of SCN1A loss-of-function variants has immediate therapeutic ramifications, as sodium channel-blocking antiseizure medications (ASMs), including carbamazepine, oxcarbazepine, lamotrigine, and phenytoin, are contraindicated owing to the risk of paradoxical seizure exacerbation and status epilepticus (Brunklaus et al., 2012; Guerrini et al., 1998). Beyond SCN1A, an expanding array of genes, including SCN1B, SCN2A, SCN8A, KCNQ2, PCDH19, and GABRG2, contributes to the genetic heterogeneity of fever-sensitive epilepsy syndromes, necessitating comprehensive multi-gene panel-based approaches for adequate diagnostic capture (Afawi et al., 2016; Kurekci et al., 2024; Myers and Mefford, 2015).

However, a critical diagnostic challenge is that the initial clinical presentation of febrile seizures is largely indistinguishable between genetically determined and non-genetic cases, and the definitive phenotypic features of syndromes such as Dravet syndrome typically manifest only after months to years of evolving seizure semiology (Harkin et al., 2007). This diagnostic latency creates a clinically consequential window during which children may receive inappropriate pharmacotherapy, potentially precipitating iatrogenic seizure aggravation (Brunklaus et al., 2012; Guerrini et al., 1998). Although recent multicenter investigations have begun to characterize SCN1A variant frequencies in large febrile seizure cohorts (Wang et al., 2025) and to elucidate genotype–phenotype correlations across functionally critical protein domains (Gallagher et al., 2024), population-based genetic screening studies employing comprehensive epilepsy gene panels in well-characterized, high-risk febrile seizure cohorts remain sparse, particularly within Chinese pediatric populations, where the majority of existing data are derived from cohorts with established epilepsy diagnoses rather than from the earlier febrile seizure stage (Chen et al., 2025; Dong et al., 2024).

Herein, we present findings from a systematic genetic screening initiative targeting high-risk febrile seizure populations in Hubei Province, China. By leveraging a validated targeted gene panel coupled with next-generation sequencing in 233 children fulfilling stringent high-risk inclusion criteria, we aimed to: (i) quantify the diagnostic yield of targeted panel-based genetic screening in a clinically stratified cohort; (ii) delineate the genotypic spectrum with respect to causal genes, variant types, inheritance patterns, and ACMG pathogenicity classifications; (iii) evaluate genotype–phenotype associations of clinical relevance; and (iv) assess the downstream therapeutic implications of molecular genetic findings.

## 2 Materials and methods

### 2.1 Study design, setting, and ethical approval

This retrospective, single-center observational study was conducted at the Department of Neurology, Wuhan Children’s Hospital (designated as the Hubei Children’s Medical Center) in collaboration with member institutions of the Hubei Pediatric Medical Alliance. The protocol was approved by the Institutional Ethics Committee of Wuhan Children’s Hospital (Approval No. 2025R059-E0). All procedures were conducted in accordance with the Declaration of Helsinki. This study is reported in adherence to the Strengthening the Reporting of Observational Studies in Epidemiology (STROBE) guidelines.

### 2.2 Study population and eligibility criteria

Children aged 6 months to 6 years presenting with documented febrile seizures were consecutively screened for enrollment between July 2019 and January 2025. Eligible patients were required to satisfy at least one of the following predefined high-risk criteria, formulated based on established prognostic indicators for epilepsy conversion (Lee et al., 2016; Sartori et al., 2019; Tsai et al., 2018): (1) complex febrile seizures, defined as seizures with a duration exceeding 15 min, focal or lateralizing semiology, or recurrence of ≥ 2 episodes within 24 h; (2) abnormal interictal electroencephalographic (EEG) findings recorded ≥ 14 days after the acute seizure episode; (3) abnormal brain magnetic resonance imaging (MRI); (4) documented developmental delay; (5) positive family history of febrile seizures or epilepsy in first- or second-degree relatives; and (6) high seizure recurrence frequency (≥ 3 episodes within 6 months or ≥ 4 within 12 months).

Exclusion criteria were as follows: structural brain lesions directly causing seizures in the absence of a suspected genetic etiology; confirmed metabolic disorders causing seizures; acute symptomatic seizures attributable solely to central nervous system infection; parental refusal of genetic testing; and insufficient DNA quality or quantity for sequencing.

### 2.3 Clinical data collection and phenotyping

Comprehensive clinical data were systematically extracted from electronic medical records, encompassing: demographic characteristics (sex, age at first seizure, age at genetic testing); seizure phenotype (type, semiology, duration, frequency, history of status epilepticus defined as seizure duration ≥ 30 min or recurrent seizures without recovery of consciousness); interictal EEG findings classified as normal, nonspecifically abnormal, or epileptiform; brain MRI findings; neurodevelopmental milestones; three-generation family pedigree data; and antiseizure medication history, including changes prompted by genetic results. The available de-identified source data tables are provided in Supplementary Table 3. Phenotypic classification at last follow-up was performed in accordance with the ILAE 2017 classification framework (Scheffer et al., 2017) and syndrome-specific diagnostic criteria for Dravet syndrome (Wirrell et al., 2017) and GEFS+ (Wallace et al., 1998).

### 2.4 Genetic testing: targeted gene panel sequencing

Peripheral venous blood samples (2–4 mL in EDTA) were collected from each proband and, where available, from both biological parents for segregation analysis. Genomic DNA was extracted using the QIAamp DNA Blood Mini Kit (Qiagen, Hilden, Germany). DNA quality and concentration were verified using a NanoDrop 2000 spectrophotometer (A260/A280 ratio: 1.8–2.0; concentration ≥ 50 ng/μL). Targeted capture enrichment was performed using the N048 Epilepsy Comprehensive Gene Detection Panel (Version 2), which encompasses all established fever-sensitive epilepsy syndrome spectrum genes, including SCN1A, SCN1B, SCN2A, SCN8A, SCN9A, KCNQ2, KCNQ3, GABRG2, GABRA1, GABRB3, PCDH19, GRIN2A, GRIN2B, CACNA1A, CACNA1E, CHD2, STXBP1, STX1B, PRRT2, SLC2A1, TSC1, TSC2, CDKL5, and additional epileptic encephalopathy-associated genes. Sequencing was performed on the Illumina NovaSeq 6000 platform (paired-end 2 × 150 bp), achieving a mean sequencing depth of ≥ 100× across targeted regions with ≥ 99% of target bases covered at ≥ 20×.

### 2.5 Bioinformatic analysis and variant classification

Raw sequencing data were subjected to quality assessment (FastQC v0.11.9), adapter trimming (Trimmomatic v0.39), alignment to the GRCh37/hg19 reference genome (BWA-MEM v0.7.17), duplicate marking (Picard v2.27.4), base quality score recalibration, and variant calling (GATK v4.4 HaplotypeCaller). Copy number variation (CNV) analysis was performed using ExomeDepth v1.1.15 and CNVkit v0.9.10. Variants were annotated using ANNOVAR against RefSeq, gnomAD v3.1, ClinVar (accessed December 2024), HGMD Professional 2024.3, and *in silico* pathogenicity prediction tools (SIFT, PolyPhen-2, MutationTaster2, CADD v1.6, REVEL). Variant pathogenicity was classified in accordance with the American College of Medical Genetics and Genomics/Association for Molecular Pathology (ACMG/AMP) five-tier guidelines (Richards et al., 2015); only variants classified as pathogenic (P) or likely pathogenic (LP) were considered diagnostically positive.

### 2.6 Statistical analysis

All analyses were performed using IBM SPSS Statistics version 26.0 (IBM Corp., Armonk, NY, USA) and R version 4.3.2 (R Foundation for Statistical Computing, Vienna, Austria; packages: stats v4.3.2, exact2 * 2 v1.6.9, epitools v0.5-10.1). Statistical methods were aligned with the ACEM 2025 reporting guidelines (Kujawa et al., 2025). No formal *a priori* sample size calculation was performed, and enrollment was determined by consecutive clinical ascertainment during the study period.

Assessment of distributional assumptions: The normality of all continuous variables was evaluated prior to the selection of inferential methods. For variables with per-group sample sizes of 10–50, the Shapiro–Wilk test was employed; for those with per-group sample sizes exceeding 50, the Lilliefors (Kolmogorov–Smirnov) test was applied, supplemented by visual inspection of histograms and quantile–quantile plots. Results are reported in Supplementary Table 1. As all continuous variables exhibited statistically significant departures from normality (all *p* < 0.050), nonparametric methods were adopted throughout.

Descriptive statistics: Continuous variables are expressed as median (first quartile [Q1], third quartile [Q3]). For subgroups with sample sizes ≤ 8, the range (minimum–maximum) is reported instead of the mean. Categorical variables are presented as absolute frequencies and percentages, *n* (%).

Inferential statistics: Between-group comparisons of continuous variables employed the Mann–Whitney U test; results are reported in the format: “M–W test: *n* = (*n*1, *n*2), U = X, r_rb = X.XX, *p* = X.XXX,” where r_rb denotes the rank-biserial correlation as a nonparametric effect size measure. Categorical comparisons utilized Pearson’s χ^2^ test of independence with Yates continuity correction for all 2 × 2 contingency tables when all expected cell frequencies were ≥ 5, or Fisher’s exact test when any expected cell frequency was < 5. The applicability of the χ^2^ test was verified by computing expected cell frequencies for each comparison; all χ^2^ analyses reported herein satisfied the minimum expected frequency criterion (smallest expected cell frequency: 8.3; see Supplementary Table 2). For multi-group comparisons of continuous variables, the Kruskal–Wallis H test was employed, with Dunn’s *post hoc* pairwise comparisons and Bonferroni correction for multiplicity.

Effect sizes: Odds ratios (OR) with 95% confidence intervals (CI) were computed for all dichotomous outcomes. For 2 × 2 tables containing a zero cell, a Haldane–Anscombe correction (addition of 0.5 to each cell) was applied to permit OR estimation; such corrected estimates are annotated in the relevant tables. All *p*-values are two-sided and reported to three decimal places; *p* < 0.001 denotes values below this threshold. The significance level was set at α = 0.05. Given the exploratory nature of the subgroup analyses, no correction for multiple comparisons was applied across the primary inferential tests; accordingly, findings from small subgroup analyses (*n* < 20) should be interpreted as hypothesis-generating.

## 3 Results

### 3.1 Cohort demographics and baseline characteristics

**FIGURE 1:**
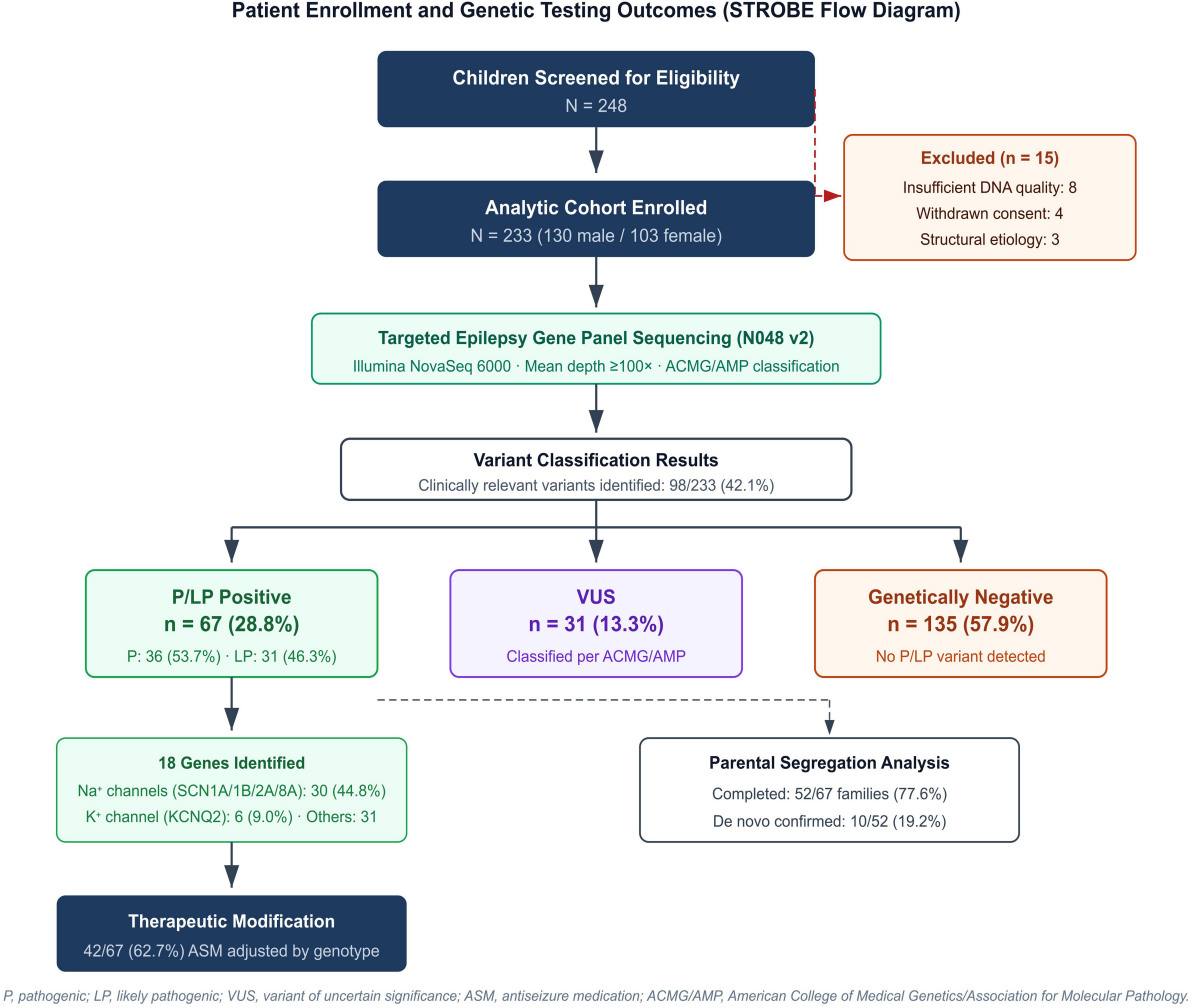
Patient enrollment and genetic testing outcomes (STROBE flow diagram). The diagram illustrates the screening-to-classification pipeline, from initial eligibility assessment (*N* = 248) through exclusions (*n* = 15) to the final analytic cohort (*N* = 233), variant classification into three categories (P/LP, VUS, and genetically negative), parental segregation analysis, and downstream therapeutic modification.

Of the 248 children screened, 233 met the eligibility criteria and comprised the final analytic cohort. Fifteen were excluded: 8 for insufficient DNA quality, 4 for withdrawn consent, and 3 for identification of a structural etiology upon further investigation (Figure 1). The cohort comprised 130 males (55.8%) and 103 females (44.2%; male-to-female ratio: 1.26:1). The median age at first seizure was 12.0 months (Q1–Q3: 7.0–24.0), and the median age at genetic testing was 26.0 months (Q1–Q3: 13.0–48.0). Complex febrile seizures constituted the most prevalent enrollment criterion (178 patients, 76.4%), followed by abnormal interictal EEG (89, 38.2%), positive family history of FS or epilepsy (62, 26.6%), documented developmental delay (41, 17.6%), abnormal brain MRI (34, 14.6%), and high seizure recurrence (29, 12.4%). Individual patients could fulfill more than one criterion; accordingly, 127 patients (54.5%) met ≥ 2 high-risk criteria simultaneously. A documented history of status epilepticus, defined as a seizure of ≥ 30 min’ duration or recurrent seizures without recovery of consciousness between events, was present in 49 patients (21.0%) at the time of enrollment. Detailed baseline characteristics stratified by genetic testing outcome are presented in Table 1.

**TABLE 1:** Demographic and clinical characteristics stratified by genetic testing outcome.

Characteristic	All (*N* = 233)	P/LP+ (*n* = 67)	Neg (*n* = 166)	Test statistic	*p*	Effect size
Male, *n* (%)	130 (55.8)	38 (56.7)	92 (55.4)	χ^2^_c = 0.001, df = 1	0.973	OR = 1.05 (0.60–1.85)
Female, *n* (%)	103 (44.2)	29 (43.3)	74 (44.6)			
Age at onset, mo, median (Q1, Q3)	12.0 (7.0, 24.0)	9.0 (5.0, 16.0)	14.0 (8.0, 26.0)	M–W: U = 4,105; *n* = (67, 166)	0.002	r_rb = 0.26
Age at testing, mo, median (Q1, Q3)	26.0 (13.0, 48.0)	22.0 (10.0, 40.0)	28.0 (14.0, 52.0)	M–W: U = 4,808; *n* = (67, 166)	0.074	r_rb = 0.14
Complex FS, *n* (%)	178 (76.4)	54 (80.6)	124 (74.7)	χ^2^_c = 0.76, df = 1	0.384	OR = 1.41 (0.71–2.80)
Abnormal EEG, *n* (%)	89 (38.2)	37 (55.2)	48 (28.9)	χ^2^_c = 13.14, df = 1	< 0.001	OR = 3.03 (1.69–5.45)
Family history, *n* (%)	62 (26.6)	20 (29.9)	42 (25.3)	χ^2^_c = 0.30, df = 1	0.584	OR = 1.25 (0.66–2.38)
Dev. delay, *n* (%)	41 (17.6)	25 (37.3)	23 (13.9)	χ^2^_c = 14.73, df = 1	< 0.001	OR = 3.70 (1.91–7.18)
Abnormal MRI, *n* (%)	34 (14.6)	14 (20.9)	20 (12.0)	χ^2^_c = 2.74, df = 1	0.098	OR = 1.93 (0.91–4.12)
High recurrence, *n* (%)	29 (12.4)	12 (17.9)	17 (10.2)	χ^2^_c = 2.22, df = 1	0.136	OR = 1.92 (0.86–4.30)
Status epilepticus, *n* (%)	49 (21.0)	28 (41.8)	21 (12.7)	χ^2^_c = 22.88, df = 1	< 0.001	OR = 4.96 (2.54–9.66)
≥ 2 high-risk criteria, *n* (%)	127 (54.5)	45 (67.2)	82 (49.4)	χ^2^_c = 5.38, df = 1	0.020	OR = 2.10 (1.16–3.79)

χ^2^_c, Pearson’s chi-square with Yates continuity correction; M–W, Mann–Whitney U test; OR, odds ratio; CI, 95% confidence interval; r_rb, rank-biserial correlation; FS, febrile seizures; EEG, electroencephalography; MRI, magnetic resonance imaging. Patients could fulfill more than one high-risk criterion; percentages for individual criteria do not sum to 100%. All expected cell frequencies were ≥ 5, confirming appropriateness of the χ^2^ test for all categorical comparisons in this table (minimum expected cell frequency: 8.3 for high seizure recurrence). Continuous variables: median (Q1, Q3).

### 3.2 Diagnostic yield

Targeted sequencing identified clinically relevant variants in 98 of 233 patients (42.1%). Of these, 67 (28.8%) harbored P/LP variants (pathogenic: *n* = 36, 53.7%; likely pathogenic: *n* = 31, 46.3%), 31 (13.3%) carried variants of uncertain significance (VUS), and 135 (57.9%) were genetically negative. The primary diagnostic yield (P/LP) was significantly higher among patients fulfilling ≥ 2 high-risk criteria than among those meeting a single criterion (45/127, 35.4% vs. 22/106, 20.8%; Pearson’s χ^2^ with continuity correction: χ^2^_c = 5.38, df = 1, *p* = 0.020; OR = 2.10, 95% CI: 1.16–3.79).

**FIGURE 2:**
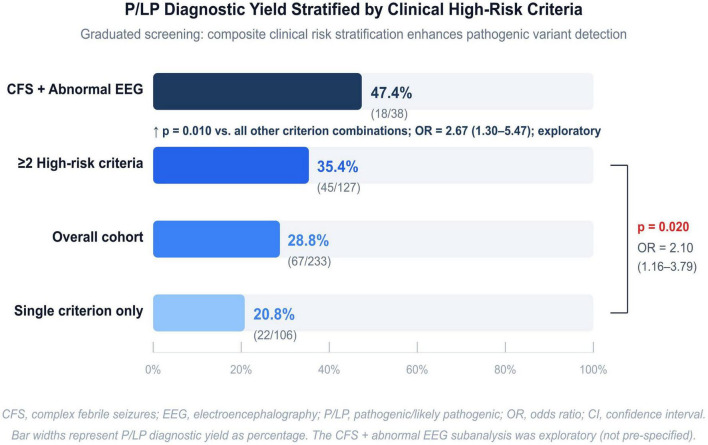
P/LP diagnostic yield stratified by clinical high-risk criteria. Horizontal bars represent the P/LP diagnostic yield across four levels of clinical risk stratification. Bar shading progresses from dark (highest yield) to light (lowest yield). The bracket with statistical annotation indicates the comparison between patients fulfilling ≥ 2 vs. a single high-risk criterion. The CFS + abnormal EEG subanalysis was exploratory (not pre-specified). CFS, complex febrile seizures; EEG, electroencephalography.

In an exploratory subgroup analysis stratified by specific criterion combinations, patients with concurrent CFS and abnormal interictal EEG demonstrated the highest P/LP yield: 18/38 (47.4%), compared with 49/195 (25.1%) for all other criterion combinations (Pearson’s χ^2^ with continuity correction: χ^2^_c = 6.67, df = 1, *p* = 0.010; OR = 2.67, 95% CI: 1.30–5.47) (Figure 2). This subgroup analysis was not pre-specified and should be interpreted as hypothesis-generating.

### 3.3 Genotypic spectrum

**FIGURE 3:**
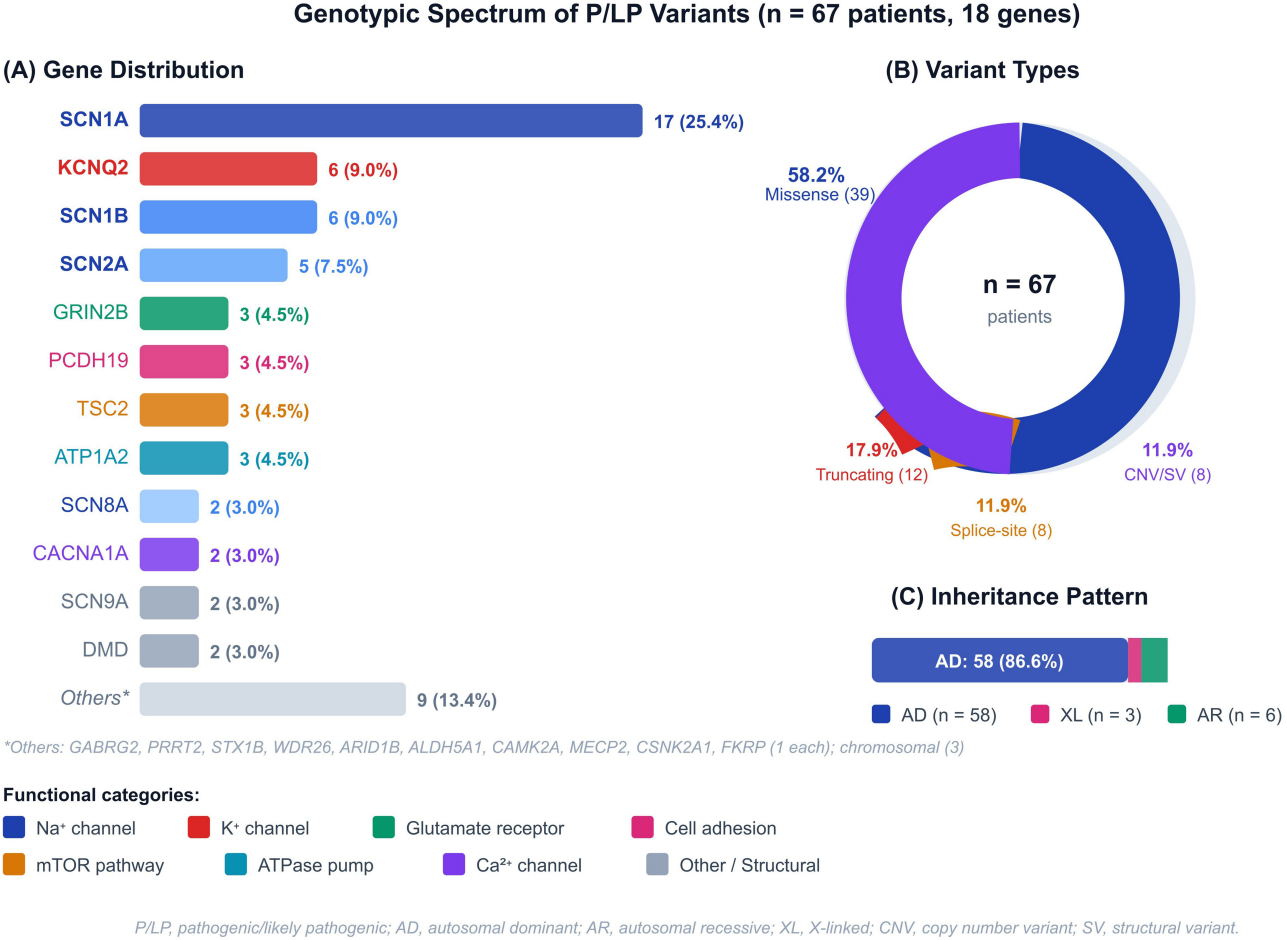
Genotypic spectrum of P/LP variants (*n* = 67 patients, 18 genes). **(A)** Horizontal bar chart of gene-level distribution among P/LP-positive patients, ranked by patient count. The bars are color-coded by functional gene category (see legend inset). **(B)** Donut chart depicting the proportional distribution of variant types (missense, protein-truncating, splice-site, and CNV/structural). **(C)** Stacked bar showing inheritance pattern distribution (autosomal dominant, autosomal recessive, and X-linked).

The 67 P/LP-positive patients harbored variants distributed across 18 distinct genes (Figure 3A and Table 2). Voltage-gated sodium channel genes central to epilepsy pathogenesis (SCN1A, SCN1B, SCN2A, and SCN8A) collectively constituted the largest functional category (30/67, 44.8%). SCN9A (*n* = 2), although encoding a voltage-gated sodium channel α-subunit, was analyzed separately, given its predominant expression in peripheral nervous system nociceptive neurons.

**TABLE 2:** Pathogenic/likely pathogenic genes identified in 67 genetically confirmed patients.

Gene	*n* (%)	Category	Variant type	ACMG	*De novo*	Syndrome(s)
SCN1A	17 (25.4)	Na^+^ channel	Missense 64.7%	12P/5LP	6/14	DS, GEFS+, DEE6B
KCNQ2	6 (9.0)	K^+^ channel	Missense 100%	4P/2LP	1/5	BFNS, DEE7
SCN1B	6 (9.0)	Na^+^ channel	Missense 100%	3P/3LP	0/5	GEFS+
SCN2A	5 (7.5)	Na^+^ channel	Miss./nonsense	3P/2LP	2/4	BFIE3, DEE11
GRIN2B	3 (4.5)	Glu receptor	Missense 100%	1P/2LP	0/2	DEE27, ID6
PCDH19	3 (4.5)	Cell adhesion	Miss./frame.	1P/2LP	1/3	DEE9
TSC2	3 (4.5)	mTOR pathway	Nonsense/splice	2P/1LP	2/3	TSC epilepsy
ATP1A2	3 (4.5)	ATPase pump	Missense 100%	0P/3LP	0/3	HM, AHC
SCN8A	2 (3.0)	Na^+^ channel	Missense 100%	0P/2LP	½	BFIE5, DEE13
Others†	19 (28.4)	Various	Various	10P/9LP	–	Various
Total	67 (100)			36P/31LP	10/52‡	

P, pathogenic; LP, likely pathogenic; DS, Dravet syndrome; GEFS+, genetic epilepsy with febrile seizures plus; DEE, developmental and epileptic encephalopathy (numerical suffixes denote OMIM-designated subtypes: DEE6B, OMIM #619317; DEE7, OMIM #613720; DEE9, OMIM #300088; DEE11, OMIM #613721; DEE13, OMIM #614558; DEE27, OMIM #616139); BFNS, benign familial neonatal seizures; BFIE, benign familial infantile epilepsy; HM, hemiplegic migraine; AHC, alternating hemiplegia of childhood; TSC, tuberous sclerosis complex. †Others: CACNA1A (2), SCN9A (2), DMD (2), GABRG2, PRRT2, STX1B, WDR26, ARID1B, ALDH5A1, CAMK2A, MECP2, CSNK2A1, FKRP (1 each), chromosomal variants (3). ‡52 families completed parental segregation.

**FIGURE 4:**
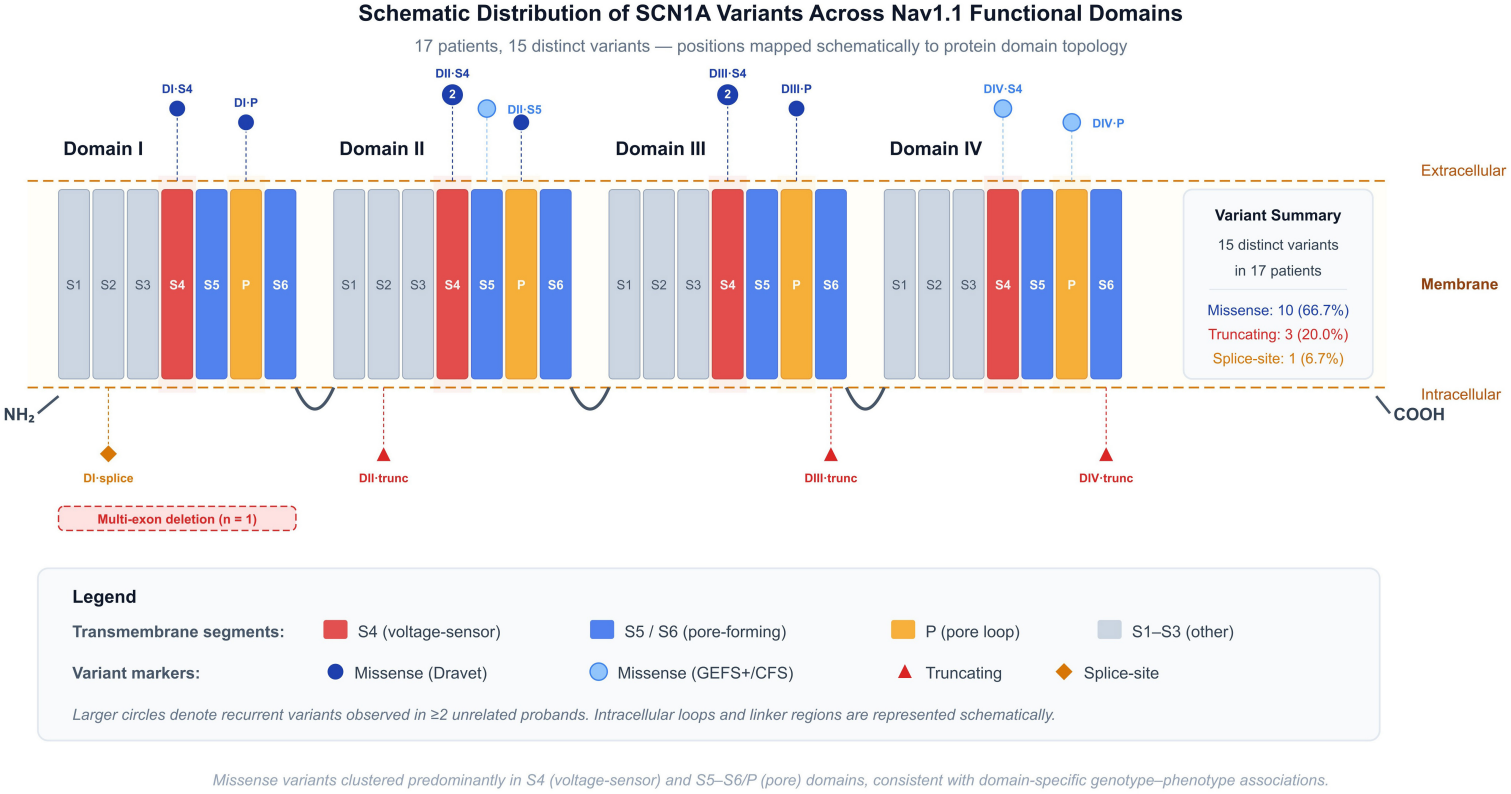
Schematic distribution of SCN1A variants across Nav1.1 functional domains. The four homologous transmembrane domains (DI–DIV) are depicted, each comprising segments S1–S6 and a pore loop (P). Functionally critical regions are color-coded as follows: S4 voltage sensor (red), S5/S6 pore-forming (blue), and pore loop (amber). Variant markers denote missense (circles; dark blue = Dravet syndrome, light blue = GEFS+/CFS), truncating (red triangles), and splice-site (amber diamonds) variants. Larger circles indicate recurrent variants in ≥ 2 unrelated probands. The dashed rectangle indicates a multi-exon deletion.

SCN1A was the single most frequently implicated gene (17 patients, 25.4% of P/LP cases), harboring 15 distinct variants, 2 of which recurred in unrelated probands. These variants comprised 10 missense substitutions (predominantly localized to the S4 voltage-sensor and S5–S6 pore-forming domains; Figure 4), 3 protein-truncating variants (2 nonsense, 1 frameshift), 1 canonical splice-site disruption, and 1 multi-exon deletion detected by read-depth-based CNV analysis. KCNQ2 was identified in 6 patients (9.0%), all missense variants affecting voltage-sensing or pore-lining regions. SCN1B was detected in 6 patients (9.0%), and SCN2A in 5 (7.5%), the latter including 2 gain-of-function variants associated with early-onset developmental and epileptic encephalopathy (DEE). SCN8A variants were identified in 2 patients (3.0%); both were heterozygous missense substitutions situated within transmembrane segments critical to channel inactivation, with one variant confirmed as *de novo* and the second of indeterminate parental origin owing to incomplete segregation analysis. Clinically, both children presented with afebrile seizures within the first six months of life and exhibited moderate developmental delay at last follow-up, a profile concordant with reported SCN8A-associated developmental and epileptic encephalopathy type 13 (DEE13). Variants in DMD were identified in 2 male patients (3.0%); both presented with prolonged febrile seizures alongside delayed motor milestones, calf pseudohypertrophy, and markedly elevated serum creatine kinase, consistent with concurrent dystrophinopathy. The seizure phenotype in these patients aligns with reports of comorbid epilepsy occurring in approximately 5–7% of children with Duchenne or Becker muscular dystrophy, an association attributed to deficiency of brain-expressed dystrophin isoforms (Dp140 and Dp71) within central nervous system neurons. Beyond ion channel genes, additional implicated loci included GRIN2B (*n* = 3), PCDH19 (*n* = 3), TSC2 (*n* = 3), ATP1A2 (*n* = 3), CACNA1A (*n* = 2), and single occurrences of GABRG2, PRRT2, STX1B, WDR26, ARID1B, ALDH5A1, CAMK2A, MECP2, CSNK2A1, and FKRP, in addition to 3 chromosomal-level variants (Table 2).

Regarding variant type across all 67 P/LP-positive cases, missense variants predominated (39/67, 58.2%), followed by protein-truncating variants (nonsense and frameshift; 12/67, 17.9%), splice-site variants (8/67, 11.9%), and copy number or structural variants (8/67, 11.9%) (Figure 3B). Most variants were heterozygous (58/67, 86.6%), consistent with autosomal dominant inheritance (Figure 3C). Hemizygous variants were identified in 2 patients (X-linked: MECP2, DMD), heterozygous variants following the X-linked cellular interference pattern were identified in 3 female patients with PCDH19, and compound heterozygous or homozygous variants consistent with autosomal recessive inheritance were present in 6 patients.

### 3.4 *De novo* variant analysis

Parental segregation analysis was performed in 52 of 67 P/LP-positive families (77.6%); the remaining 15 families were unavailable for parental testing owing to logistical constraints (*n* = 9) or incomplete sample collection (*n* = 6). *De novo* origin was confirmed in 10 of 52 assessable families (19.2% of those with completed segregation; 14.9% of all 67 P/LP-positive patients), predominantly involving SCN1A (6/10, 60.0%) and SCN2A (2/10, 20.0%). Among *de novo* SCN1A carriers, 5 of 6 (83.3%) progressed to Dravet syndrome.

Compared with patients harboring inherited P/LP variants (*n* = 42), those with confirmed *de novo* variants (*n* = 10) exhibited significantly higher rates of developmental regression (7/10, 70.0% vs. 11/42, 26.2%; Fisher’s exact test: *p* = 0.017; OR = 6.53, 95% CI: 1.42–30.04), drug-resistant epilepsy (5/10, 50.0% vs. 7/42, 16.7%; Fisher’s exact test: *p* = 0.043; OR = 5.00, 95% CI: 1.10–22.73), Dravet syndrome diagnosis (5/10, 50.0% vs. 7/42, 16.7%; Fisher’s exact test: *p* = 0.043; OR = 5.00, 95% CI: 1.10–22.73), and seizure onset before 9 months of age (8/10, 80.0% vs. 16/42, 38.1%; Fisher’s exact test: *p* = 0.029; OR = 6.50, 95% CI: 1.21–34.88) (Table 3). The wide confidence intervals associated with these estimates reflect the limited sample size of the *de novo* subgroup, and these findings should be interpreted as hypothesis generating.

**TABLE 3:** Clinical outcomes in patients with *de novo* versus inherited P/LP variants (*n* = 52 with completed parental segregation).

Feature	*De novo* (*n* = 10)	Inherited (*n* = 42)	Test; *p*	OR (95% CI)
Developmental regression, *n* (%)	7 (70.0)	11 (26.2)	Fisher: *p* = 0.017	6.53 (1.42–30.04)
Drug-resistant epilepsy, *n* (%)	5 (50.0)	7 (16.7)	Fisher: *p* = 0.043	5.00 (1.10–22.73)
Dravet syndrome, *n* (%)	5 (50.0)	7 (16.7)	Fisher: *p* = 0.043	5.00 (1.10–22.73)
Seizure onset < 9 months, *n* (%)	8 (80.0)	16 (38.1)	Fisher: *p* = 0.029	6.50 (1.21–34.88)

Fisher, Fisher’s exact test. OR, odds ratio; CI, confidence interval. Wide confidence intervals reflect the limited sample sizes; these findings should be regarded as hypothesis-generating.

To address the phenotypic spectrum among carrier parents of inherited variants, segregation data from the 42 families harboring confirmed inherited P/LP variants were systematically reviewed. A documented history of childhood febrile seizures was identified in 16 carrier parents (38.1%), the majority of which had presented as simple febrile seizures with spontaneous resolution by 5–6 years of age and without progression to afebrile epilepsy. Seven carrier parents (16.7%) exhibited GEFS+ spectrum manifestations, including febrile seizures plus or afebrile seizures persisting into adolescence, and 5 (11.9%) carried an established epilepsy diagnosis with sustained seizure freedom on monotherapy at the time of assessment. The remaining 14 carrier parents (33.3%) were neurologically asymptomatic and had no documented seizure history despite molecular confirmation of the variant. Phenotypic variability was particularly pronounced within SCN1B kindreds (5 inherited families), in which parental phenotypes ranged from asymptomatic carriage to clinically defined GEFS+, and in PCDH19 families, where heterozygous-carrier mothers were predominantly subclinical, consistent with the X-linked cellular interference model of pathogenesis. These observations corroborate the reduced penetrance and variable expressivity that characterize many fever-sensitive epilepsy kindreds and carry important implications for genetic counseling regarding recurrence risk estimation and prognostic prediction in affected families.

### 3.5 Genotype–phenotype correlations

#### 3.5.1 SCN1A-positive patients

**FIGURE 5:**
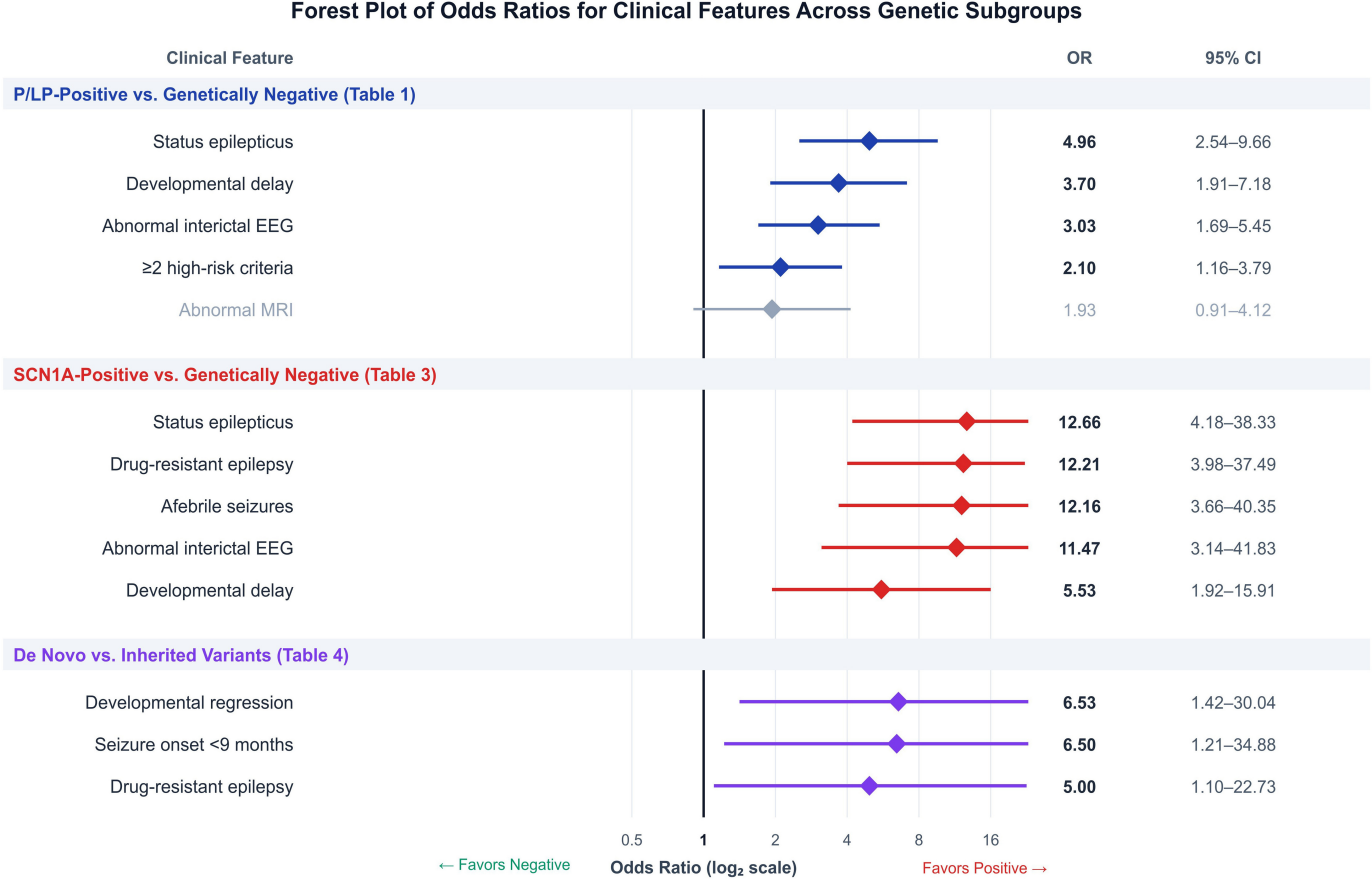
Forest plot of odds ratios for clinical features across genetic subgroups. Odds ratios (diamonds) with 95% confidence intervals (horizontal lines) are displayed on a log2 scale across three comparison strata: P/LP-positive vs. genetically negative (blue; Table 1), SCN1A-positive vs. genetically negative (red; Table 4), and *de novo* vs. inherited variants (purple; Table 3). The vertical line at OR = 1 denotes the null. Gray symbols indicate nonsignificant associations (95% CI crossing 1).

Among 17 SCN1A-positive patients, phenotypic classification at last follow-up revealed Dravet syndrome in 12 (70.6%), GEFS+ in 3 (17.6%), and CFS without progression to established epilepsy in 2 (11.8%). Compared with the genetically negative patients (*n* = 166), SCN1A-positive patients exhibited markedly elevated prevalence of status epilepticus (11/17, 64.7% vs. 21/166, 12.7%; Fisher’s exact test: *p* < 0.001; OR = 12.66, 95% CI: 4.18–38.33), developmental delay (8/17, 47.1% vs. 23/166, 13.9%; Fisher’s exact test: *p* = 0.002; OR = 5.53, 95% CI: 1.92–15.91), abnormal interictal EEG (14/17, 82.4% vs. 48/166, 28.9%; Fisher’s exact test: *p* < 0.001; OR = 11.47, 95% CI: 3.14–41.83), afebrile seizures (13/17, 76.5% vs. 35/166, 21.1%; Fisher’s exact test: *p* < 0.001; OR = 12.16, 95% CI: 3.66–40.35), and drug-resistant epilepsy (9/17, 52.9% vs. 14/166, 8.4%; Fisher’s exact test: *p* < 0.001; OR = 12.21, 95% CI: 3.98–37.49) (Figure 5 and Table 4). Fisher’s exact test was applied throughout these comparisons, as the small SCN1A-positive subgroup (*n* = 17) produced expected cell frequencies below 5 in multiple cells. The Dravet syndrome comparison (12/17 vs. 0/166) yielded *p* < 0.001 by Fisher’s exact test; an OR could not be directly computed owing to the zero cell in the genetically negative group.

**TABLE 4:** Clinical features of SCN1A-positive patients versus genetically negative patients.

Feature	SCN1A+ (*n* = 17)	Negative (*n* = 166)	Test; *p*	OR (95% CI)
Status epilepticus, *n* (%)	11 (64.7)	21 (12.7)	Fisher: *p* < 0.001	12.66 (4.18–38.33)
Developmental delay, *n* (%)	8 (47.1)	23 (13.9)	Fisher: *p* = 0.002	5.53 (1.92–15.91)
Abnormal interictal EEG, *n* (%)	14 (82.4)	48 (28.9)	Fisher: *p* < 0.001	11.47 (3.14–41.83)
Afebrile seizures, *n* (%)	13 (76.5)	35 (21.1)	Fisher: *p* < 0.001	12.16 (3.66–40.35)
Drug-resistant epilepsy, *n* (%)	9 (52.9)	14 (8.4)	Fisher: *p* < 0.001	12.21 (3.98–37.49)
Dravet syndrome, *n* (%)	12 (70.6)	0 (0.0)	Fisher: *p* < 0.001	—§

Fisher, Fisher’s exact test (applied throughout owing to expected cell frequencies < 5 given the small SCN1A+ subgroup). OR, odds ratio; CI, confidence interval; EEG, electroencephalography. §OR not directly estimable owing to a zero cell in the genetically negative group. With Haldane–Anscombe correction (0.5 added to each cell): OR ≈ 461.5 (95% CI: 25.6–8,317); this estimate should be interpreted with extreme caution given its instability. *p*-values reported to three decimal places per ACEM guidelines (Kujawa et al., 2025).

#### 3.5.2 KCNQ2-positive patients

The 6 KCNQ2-positive patients presented a phenotypically distinct profile with neonatal or early infantile seizure onset (median: 8 days; range: 1–90 days). The predominant seizure semiology comprised tonic posturing with bilateral eye deviation. Interictal EEG patterns included burst suppression (*n* = 2), multifocal sharp-wave discharges (*n* = 3), and focal rhythmic discharges (*n* = 1). Four patients were classified as self-limited (benign) familial neonatal epilepsy (SeLFNE), all of whom achieved seizure remission with carbamazepine monotherapy, whereas 2 patients met criteria for neonatal-onset DEE with ongoing seizures despite polytherapy.

#### 3.5.3 Comparison across major genetic subgroups

To contextualize phenotypic differences across the three largest subgroups, SCN1A-positive (*n* = 17), KCNQ2-positive (*n* = 6), and genetically negative (*n* = 166), selected clinical variables were compared using multi-group nonparametric tests. Statistically significant between-group differences were observed for median age at seizure onset (Kruskal–Wallis H test: H = 26.38, df = 2, *p* < 0.001; Dunn’s *post hoc* with Bonferroni correction: SCN1A-positive vs. genetically negative, adjusted *p* < 0.001; KCNQ2-positive vs. genetically negative, adjusted *p* = 0.003; SCN1A-positive vs. KCNQ2-positive, adjusted *p* = 0.412), proportion with developmental delay (Fisher–Freeman–Halton exact test: *p* = 0.002), and proportion achieving seizure freedom on monotherapy (Fisher–Freeman–Halton exact test: *p* < 0.001). Given the limited sample size in the KCNQ2 subgroup (*n* = 6), these multigroup comparisons should be interpreted with appropriate caution.

### 3.6 Therapeutic implications

Genetic findings directly influenced ASM management in 42 of 67 (62.7%) P/LP-positive patients. Therapeutic modifications encompassed three categories: avoidance or discontinuation of contraindicated medications (23/67, 34.3%), targeted initiation of gene-specific pharmacotherapy (14/67, 20.9%), and referral for pathway-specific or investigational therapy (5/67, 7.5%).

Among the SCN1A-positive patients (*n* = 17), sodium channel-blocking agents were avoided or discontinued in all cases; first-line therapy was redirected to valproate, levetiracetam, or clobazam. Notably, of the 8 SCN1A-positive patients who had received sodium channel blockers prior to the availability of genetic results, 6 (75.0%) experienced documented seizure aggravation that resolved upon medication substitution. KCNQ2-SeLFNE patients (*n* = 4) demonstrated a favorable response to carbamazepine. TSC2-positive patients (*n* = 3) were evaluated for mechanistic target of rapamycin (mTOR) inhibitor therapy; one patient demonstrated a marked reduction in seizure burden following everolimus initiation. Additional actionable findings included STX1B (levetiracetam initiation), PRRT2 (carbamazepine with sustained seizure freedom), and ALDH5A1 (vigabatrin considered for succinic semialdehyde dehydrogenase deficiency).

## 4 Discussion

The present investigation constitutes one of the largest systematic genetic screening studies targeting clinically stratified high-risk febrile seizure populations in a Chinese pediatric cohort, and its principal findings have implications for diagnostic strategy, prognostic assessment, and therapeutic decision-making in this prevalent childhood condition. Four salient observations emerge: a diagnostically significant pathogenic/likely pathogenic variant detection rate of 28.8%, substantially exceeding that reported in unselected febrile seizure populations; a genotypic spectrum dominated by voltage-gated sodium and potassium channel genes; clinically robust genotype–phenotype associations demonstrating the prognostic significance of specific gene–variant combinations; and direct therapeutic actionability of genetic results in approximately two-thirds of positive patients.

The 28.8% P/LP detection rate observed in this cohort compares favorably with and must be interpreted in the context of contemporaneous studies that differ substantially in their ascertainment strategies and population characteristics. Wang et al. (2025), in a multicenter cohort of 2,552 children with febrile seizures, reported an overall pathogenic SCN1A variant rate of 5.5%, rising to 6.8% in the CFS subgroup; however, that study employed single-gene (SCN1A) screening rather than a comprehensive multi-gene panel, thereby precluding detection of non-SCN1A genetic etiologies. de Sainte Agathe et al. (2025), in a large French multicenter cohort of 2,563 epilepsy patients referred for genetic diagnosis, reported a 27.0% P/LP rate using gene panels encompassing a 68-gene core set, a figure closely aligned with our finding—and observed that diagnostic yield was highest among Dravet syndrome spectrum and early infantile DEE phenotypes. Chen et al. (2025), studying 133 Chinese GEFS+ families using whole-exome or whole-genome sequencing, reported a diagnostic yield of 23.6%, with voltage-gated channel genes and GABA receptor-related genes constituting the most commonly implicated categories. The substantially higher yield in our study relative to the unselected cohort of Wang et al. (2025) can be most parsimoniously attributed to the application of stringent multicriteria clinical risk stratification, which enriched the analytic sample for genetically determined cases. Our observation that patients fulfilling ≥ 2 high-risk criteria demonstrated a significantly higher diagnostic yield (35.4% vs. 20.8%; *p* = 0.020; OR = 2.10) provides empirical support for a graduated screening approach in resource-constrained settings. The further finding, albeit from an exploratory subgroup analysis that the concurrent presence of CFS and abnormal interictal EEG conferred the highest P/LP yield (47.4%) suggests that specific criterion combinations may refine the prioritization of patients for genetic testing, a hypothesis warranting prospective validation.

The conceptual rationale underpinning our high-risk stratification approach finds important reinforcement in the recent reappraisal by Falsaperla et al. (2023), who, drawing on a substantial pediatric cohort, have challenged the historical 15-min threshold that has long demarcated simple from complex febrile seizures and have proposed a markedly shorter operational cutoff of approximately 5 min. Their analysis suggests that febrile seizures persisting beyond this briefer interval already convey a heightened prognostic significance, an observation that resonates with the diagnostic yield gradient demonstrated in the present series. Although the revised duration threshold proposed by Falsaperla et al. (2023) has yet to attain consensus endorsement within international classification frameworks, its conceptual implication is that the boundary between “simple” and “complex” febrile seizures may be biologically more porous than the categorical ILAE definitions imply, with even modestly prolonged events potentially heralding distinct pathophysiological substrates. This perspective is concordant with our finding that an enrichment strategy capturing the prolonged-duration end of the febrile seizure spectrum, in combination with abnormal EEG findings, family history, developmental concerns, and recurrence, substantially augments the detection of clinically actionable monogenic etiologies. Should subsequent multicenter studies validate the abbreviated duration threshold proposed by Falsaperla et al. (2023), our high-risk algorithm could be readily adapted to incorporate seizure duration as a refined continuous predictor rather than a categorical one, thereby further enhancing its diagnostic sensitivity for fever-sensitive epilepsy genes.

The identification of SCN1A variants in 25.4% of genetically positive patients is consistent with the established importance of this gene in the molecular architecture of fever-sensitive epilepsy syndromes. Gallagher et al. (2024), in a landmark analysis of 1,018 individuals with SCN1A-related epilepsies, demonstrated that missense variants localized to functionally critical domains—particularly the voltage-sensing S4 segment and the pore-forming S5–S6 linker—were associated with earlier seizure onset and a higher probability of Dravet syndrome, and further established that status epilepticus as the initial seizure type exhibited high specificity (95.2%) but limited sensitivity (32.7%) for the Dravet phenotype. The present data corroborate these domain-specific associations: the majority of SCN1A missense variants in our cohort mapped to S4–S6 regions, and the Dravet syndrome progression rate among SCN1A-positive patients was 70.6%. The statistical associations between SCN1A-positive status and adverse clinical features—status epilepticus (odds ratio [OR] = 12.66), developmental delay (OR = 5.53), abnormal interictal electroencephalography (EEGs; OR = 11.47), afebrile seizures (OR = 12.16), and drug-resistant epilepsy (OR = 12.21)—underscore the prognostic value of early SCN1A identification, although the wide confidence intervals attendant upon the limited subgroup size (*n* = 17) mandate confirmation in larger cohorts. Dong et al. (2024), in a Chinese epilepsy cohort of 691 patients, similarly reported that voltage-gated sodium channel variants affecting the pore region were associated with more frequent cluster seizures and that statistically significant clinical disparities existed between SCN1A-Dravet syndrome and SCN1A-GEFS+ subgroups with respect to seizure onset age, status epilepticus incidence, and ASM responsiveness. Kurekci et al. (2024) further demonstrated the utility of functional prediction algorithms in delineating variant-level effects on sodium channel function and their correlation with phenotypic severity.

The clinical urgency of early genetic diagnosis is underscored by the iatrogenic seizure aggravation documented in this cohort: 6 of 8 SCN1A-positive patients (75.0%) who had received sodium channel-blocking ASMs prior to the availability of genetic results experienced clinically significant deterioration that resolved upon medication substitution. This observation is mechanistically coherent with the established pathophysiology of SCN1A haploinsufficiency, wherein reduced Nav1.1-mediated sodium current selectively impairs the excitability of GABAergic inhibitory interneurons, and further pharmacological sodium channel blockade exacerbates the resultant excitatory–inhibitory imbalance (Yu et al., 2006). Analogous observations regarding suboptimal pharmacotherapeutic response patterns have been reported in Taiwanese and Polish SCN1A cohorts (Stawicka et al., 2024; Syu et al., 2024), and broader SCN1A cohort characterization in Turkish patients further supports marked phenotypic heterogeneity across Dravet syndrome and GEFS+ (Türkyılmaz et al., 2022). Notably, emerging evidence for novel therapeutic modalities in the broader SCN1A-related epilepsy spectrum has been provided by Dell’Isola et al. (2026), who demonstrated that adjunctive fenfluramine achieved a mean seizure frequency reduction of 91% in 11 patients with SCN1A-related GEFS+, illustrating the expanding therapeutic landscape shaped by precision molecular diagnosis (Wirrell, 2016).

An important contribution of this investigation is the delineation of the non-SCN1A genotypic landscape. KCNQ2, identified in 9.0% of P/LP-positive cases, represents a clinically distinctive entity with a well-characterized bimodal phenotypic distribution: self-limited familial neonatal epilepsy at the benign end versus severe neonatal-onset DEE at the other (Cornet et al., 2018). The favorable response of SeLFNE-associated KCNQ2 patients to carbamazepine, a drug contraindicated in SCN1A-associated disorders, illustrates the gene-specific nature of ASM selection and the potential for iatrogenic harm when treatment is guided by syndromic classification alone rather than by molecular diagnosis. SCN1B variants, present in an equivalent proportion (9.0%) of positive cases, co-segregated with febrile seizure phenotypes across multiple family members, consistent with the autosomal dominant inheritance and variable expressivity characteristic of GEFS+ kindreds (Oliver et al., 2024; Wallace et al., 1998). The breadth of our genotypic spectrum, spanning 18 genes encompassing ion channels, glutamate receptors, cell adhesion molecules, and intracellular signaling pathway components, mirrors the genetic heterogeneity reported in the French EpiGene network, where a 68-gene core panel captured the vast majority of positive diagnoses (de Sainte Agathe et al., 2025), reinforcing the conclusion that comprehensive panel-based approaches are indispensable for adequate diagnostic capture in fever-sensitive epilepsy populations.

The confirmation of *de novo* origin in 19.2% of P/LP-positive families with completed parental segregation and the association of *de novo* status with significantly worse clinical outcomes, including higher rates of developmental regression (OR = 6.53) and drug-resistant epilepsy (OR = 5.00), are concordant with the broader epilepsy genetics literature. The Epi4K Consortium established that *de novo* variants account for a substantial fraction of severe epileptic encephalopathies (Epi 4K Consortium et al., 2013), and our findings extend this observation to the febrile seizure population. However, the limited *de novo* subgroup size (*n* = 10) and attendant wide confidence intervals necessitate circumspect interpretation of these associations as hypothesis-generating rather than definitive. Notwithstanding these statistical caveats, the distinction between *de novo* and inherited variants has fundamental implications for genetic counseling: *de novo* status effectively reduces the sibling recurrence risk to the population background rate, whereas inherited variants confer a 50% transmission probability in autosomal dominant kindreds, a distinction of considerable clinical importance that supports routine parental segregation analysis wherever logistically feasible.

The therapeutic actionability of genetic findings, documented in 62.7% of P/LP-positive patients, constitutes one of the most clinically impactful observations of this study. Ozcan et al. (2025), in a cohort of 128 neonatal/infantile-onset genetic epilepsy patients, reported a comparable therapeutic yield of 61.7% following molecular diagnosis, while Kanmaz et al. (2024), in a nationwide Turkish cohort of 1,450 early-onset DEE patients, documented potential therapeutic modifications in 56.2% of genetically confirmed cases. The convergence of these estimates across diverse populations and clinical contexts attests to the substantial therapeutic dividend conferred by molecular genetic diagnosis in pediatric epilepsy.

Several limitations warrant explicit acknowledgment. First, the retrospective single-center design introduces potential selection and referral biases inherent to a tertiary pediatric neurology service, which may limit generalizability notwithstanding the broad catchment area of the Hubei Pediatric Medical Alliance network. Second, the targeted gene panel approach does not interrogate deep intronic variants, regulatory mutations, or structural variants beyond the resolution of read-depth-based copy number variation (CNV) calling; whole-genome sequencing may identify additional pathogenic variants not captured by this methodology. Third, functional validation of novel variants was not undertaken, and current variant classifications remain subject to reclassification as additional evidence accrues. Fourth, the heterogeneous follow-up duration (median, 18 months; range, 3–68 months) may have resulted in incomplete phenotypic ascertainment, particularly for patients currently classified as having CFS without epilepsy progression, in whom longer observation may reveal evolving phenotypes. Fifth, no formal *a priori* sample size calculation was performed, and the limited sizes of certain genetic subgroups, notably the potassium voltage-gated ion channel subfamily Q member 2 (KCNQ2; *n* = 6) and *de novo* (*n* = 10) subgroups, constrained the statistical power and precision of the corresponding effect size estimates, as reflected in the wide confidence intervals observed. Sixth, given the multiplicity of statistical comparisons performed and the absence of a correction for multiple testing, the subgroup-level analyses should be regarded as exploratory and hypothesis-generating. Finally, multicenter validation encompassing geographically and ethnically diverse populations, including both Han and ethnic minority Chinese cohorts, is required to establish the external validity of these findings.

## 5 Conclusion

This study demonstrates that targeted genetic screening in clinically stratified high-risk pediatric febrile seizure populations yields a diagnostically meaningful P/LP variant detection rate of 28.8%, revealing a genotypic spectrum dominated by voltage-gated ion channel genes, principally SCN1A, KCNQ2, and SCN1B. Composite clinical risk stratification, particularly the combination of complex febrile seizures and abnormal interictal EEG, enhances diagnostic yield and supports a graduated screening strategy amenable to resource-limited settings. Robust genotype–phenotype correlations, including the prognostic significance of SCN1A variants for Dravet syndrome progression, the association of *de novo* variants with adverse clinical trajectories, and the gene-specific determinism of ASM response, furnish compelling evidence for the integration of early molecular profiling into the clinical evaluation algorithm for pediatric complex febrile seizures. Prospective multicenter investigations incorporating whole-genome approaches, standardized longitudinal outcome tracking, and health-economic analyses are warranted to refine risk stratification frameworks and quantify the clinical and economic value of early genetic testing in this population.

## Data Availability

The de-identified minimal dataset supporting the conclusions of this article is provided in the Supplementary material as Supplementary Tables S3A–S3E. These files include the English-only de-identified patient-level and variant-level source data used for the analyses, together with a README/data dictionary and curation log. Additional raw clinical records, administrative records, and sequencing-level data are not publicly available because they involve pediatric human genetic data and institutional/ethical privacy restrictions, and the authors do not have permission to publicly release data beyond the de-identified supplementary dataset. Requests for access to additional restricted data may be directed to the corresponding author(s) and will be considered only subject to institutional ethics approval, applicable data-protection requirements, and appropriate data-sharing agreements.
