# Correction: The genotypic spectrum of complex febrile seizures: insights from high-risk population genetic screening in a pediatric cohort

**DOI:** 10.3389/fnins.2026.1927983

**Published:** 2026-07-23

**Authors:** Xiaolong Deng, Yan Xu, Xue Chen, Juan Pan, Sheng Huang, Dan Sun

**Affiliations:** 1 Department of Neurology, Wuhan Children's Hospital, Tongji Medical College, Huazhong University of Science and Technology, Wuhan, Hubei, China; 2 Department of Child Health Care, Wuhan Jiang'an District Maternal and Child Health Hospital, Wuhan, Hubei, China

**Keywords:** complex febrile seizures, Dravet syndrome, genotype–phenotype correlation, ion channelopathy, pediatric epilepsy, precision medicine, SCN1A, targeted gene panel sequencing

An incorrect grant number was provided for the Hubei Provincial Natural Science Foundation of China. In the published article, the grant number was stated as **2025AF009**. The correct grant number is **2025AFC099**.

The original version of this article has been updated.

