# Supplementary material for: The genotypic spectrum of complex febrile seizures: insights from high-risk population genetic screening in a pediatric cohort
Source: Front Neurosci. 2026 Jun 5;20:1828448. doi: 10.3389/fnins.2026.1828448 (PMC13280790; doi:10.3389/fnins.2026.1828448)
Supplement: Supplementary file 1 [file Supplementary_file_1.docx]

**Supplementary Table S1.** Results of normality testing for continuous variables.

| Variable | Group | n | Test | Statistic | p-value | Normal? |
| --- | --- | --- | --- | --- | --- | --- |
| Age at onset (mo) | P/LP+ | 67 | Lilliefors | D = 0.142 | 0.002 | No |
|  | Negative | 166 | Lilliefors | D = 0.168 | <0.001 | No |
| Age at testing (mo) | P/LP+ | 67 | Lilliefors | D = 0.124 | 0.013 | No |
|  | Negative | 166 | Lilliefors | D = 0.151 | <0.001 | No |
| Total seizure count | P/LP+ | 67 | Lilliefors | D = 0.203 | <0.001 | No |
|  | Negative | 166 | Lilliefors | D = 0.187 | <0.001 | No |
| Age at onset—SCN1A+ | SCN1A+ | 17 | Shapiro–Wilk | W = 0.891 | 0.048 | No |
| Age at onset—KCNQ2+ | KCNQ2+ | 6 | —* | — | — | N/A* |

*W, Shapiro–Wilk statistic; D, Lilliefors statistic. *n < 10: normality testing unreliable per ACEM Table 1 recommendation [28]; nonparametric methods used.*

**Supplementary Table S2** . Expected cell frequencies for all Pearson's χ² tests in Table 1.

| Variable | Min. expected cell frequency | χ² appropriate? |
| --- | --- | --- |
| Sex | 29.6 | Yes |
| Complex FS | 15.8 | Yes |
| Abnormal EEG | 25.6 | Yes |
| Family history | 17.8 | Yes |
| Developmental delay | 11.8 | Yes |
| Abnormal MRI | 9.8 | Yes |
| High recurrence | 8.3 | Yes |
| Status epilepticus | 14.1 | Yes |
| ≥2 high-risk criteria | 30.5 | Yes |

*Expected cell frequencies calculated as (row total × column total) / N for each 2 × 2 table. Minimum expected cell frequency reported. The χ² test with continuity correction is considered appropriate when all expected cell frequencies are ≥5.*
